# Correlation of Different Electrical Parameters of Solar Cells with Silver Front Electrodes

**DOI:** 10.3390/ma12030366

**Published:** 2019-01-24

**Authors:** Małgorzata Musztyfaga-Staszuk, Damian Janicki, Piotr Panek

**Affiliations:** 1 Welding Department, Silesian University of Technology, Konarskiego 18A, 44-100 Gliwice, Poland; Damian.Janicki@polsl.pl; 2 Institute of Metallurgy and Materials Science PAS. Reymonta 25, 30-059 Krakow, Poland; p.panek@imim.pl

**Keywords:** front electrode, silicon solar cells, TLM method

## Abstract

This work presents comparison results of the selected electrical parameters of silicon solar cells manufactured with silver front electrodes which were co-fired in an infrared belt furnace in the temperature range of 840–960 °C. The commercial paste (PV19B) was used for the metallization process. Electrical properties of a batch of solar cells fabricated in one cycle were investigated. Three methods were used, including measurement of the current-voltage characteristics (I-V), measurement of contacts’ resistivity using the transmission Line model method (TLM), and measurement of contacts’ resistivity using the potential difference method (PD). This work is focused on both the different metallization temperatures of co-firing of solar cells and measurements using the above-mentioned methods. It is shown that the solar cell parameters measured with three methods have different, but strongly correlated values. Moreover, the comparative analysis was performed of the investigations of the same photovoltaic solar cells using both the TLM method and independent research stands (including one non-commercial and two commercial ones) at three different scientific units. In the PD and TLM methods, the same calculation formulae are used. It can be stated, comparing methods I-V, PD, and TLM, that for each, different parameters are determined to assess the electrical properties of the solar cell.

## 1. Introduction

Based on the available literature [1,2,3,4,5], we can evaluate the current status of several methods which are used for the measurement of the selected electrical parameters of a solar cell using different devices. It is generally known that most of them are destructive (such as the transmission line model (TLM), the potential difference (PD), and the current-voltage characteristics (I-V) methods) or nondestructive (for instance cTLM, known as the circular transmission line method), but require the fabrication of exceptional metal contacts. The 1960s are considered to be the period where a separate branch of related technology was created (developing interconnecting electronic components, their methods, and applications). More publications appeared in the 1990s, and more various methods were used, including, for instance, I-V and TLM among others to determine the series resistance RS of solar cells and their experimental investigation to find the most reliable and robust method(s) for cell characterization under operating conditions. The PD method is to more recent, with its first use starting in 2000.

The dopant sources may be used in solar cell production being deposited onto the wafer surface by means of screen-printing [6,7], spray-on method [8,9], or formed from the gaseous phase [10]. The contact between the electrode and the semiconductor may also depend on the type of the dopant source, which should also be taken into account when analyzing the measurement results. The effect of the selection of the metallization parameters of the electrical contacts of the cell is reflected by the result of measuring the light current-voltage characteristics (I-V), including fill factor (*FF*) and series cell resistance (*R_s_*). The current-voltage characteristics (I-V) method is widely applied and has been developed to give the opportunity to assess the correctness of the process and the cell classification based on their electrical parameters [6,11]. The most important parameter of the solar cell is its photovoltaic conversion efficiency *E_ff_*. It is the value calculated at the maximum power point from the clear I-V characteristic of the cell solar, described by dependent parameters *E_ff_*= *f* (*I_sc_*, *V_oc_*, *FF*), among which the FF fill coefficient is the function of *FF* = *f* (*R_s_*, *R_sh_*). The above parameters are determined by fundamental and technological factors, the first of which result from the nature of the c-Si material, while the second includes losses classified as electrical and optical [12]. 

The I-V measurement device is commonly known and used in scientific units. The biggest advantage of this method is that it is nondestructive. Moreover, the real temperature measurement of the Si wafer is possible during the metallization process for which the Datapaq-Solarpag system is used. It passes through the IR furnace together with the Si plate in the desired time interval and measures the plate temperature in up to six different locations. However, its cost can be disadvantageous, and the device has to be repaired while in use. Moreover, for proper operation of the IR furnace, it is necessary to have the post-reaction gas extraction system, as well as a system supplying it with working gases, which may be the natural atmosphere after purification and drying, or/and nitrogen and oxygen. The actual temperature of the cell electrode metallization process must be controlled depending on the temperature of the individual subsequent heating zones of the furnace and belt speed. The temperature profile of the metallization process must be adjusted in so that the Ag paste melts the TiO_2_ or Si_x_N_y_: H anti-reflective layer and creates a stop with the upper layer of the Si-emitter region but does not fully diffuse into the space charge region.

The electrical parameters of the electrode paths of the solar cell after the metallization process in the IR furnace [13] can be analyzed more precisely using measurements of the resistance and specific resistance. The silicon wafer connection zone in the contact structure plays the essential role at the front electrode substrate, whose resistivity can be measured using the transmission line model method (TLM) or the potential difference method (PD) [14,15,16,17]. Although the TLM method has been known for a long time, its practical use for measuring the resistance of the front electrode of silicon photovoltaic cells is more recent. The possibilities of the transmission line method and the insufficient hardware base are the reasons for the great interest in the subject measurements of various research centers, services improving the electrical properties of silicon solar cells, and the designers of measuring instruments. Therefore, in 2010, at the Institute of Engineering Materials and Biomedical Engineering Faculty of the Silesian University of Technology, the original concept of a measuring system for measuring the resistance of the front electrode with the transmission line method was proposed. The presented concept of the measurement system was used in the design of the measuring station [18,19]. Due to the fact that the author is a co-creator of the invention, for the purposes of this work, she implemented the above-mentioned measuring stand.

The author was also obliged to design and prepare an additional dedicated measuring matrix, the task of which is to deliver and output a measuring signal to and from the measurement object by means of probes in the measuring stand. The developed proprietary measurement stand allows for testing the resistance of the zone connecting the front electrode with the silicon substrate using the TLM method and serves as an alternative to the unattractive market offer of measuring instruments. Two years later, the same institute purchased new equipment, including the modern Corescan device with software for testing the electrical properties of wafers and photovoltaic cells. There are some advantages and disadvantages in the case of the TLM model method. The advantages are as follows: The costs of designing and manufacturing the matrix for measurements of the front electrode of the solar cell are low; the stand takes up little space in the room; it is cheap to repair its individual parts, and it offers the possibility of designing any pattern for the front electrode. The disadvantages are as follows: It is a destructive method and requires designing a pattern applied to the front metallization (i.e., a series of parallel lines of different sizes and distances), which at the same time involves the design of a new matrix, and consequently the cost of manufacturing this matrix. Although they are small, it is time-consuming because it does not have the complete operating software, which allows the automatic measurement of parameters with the possibility of saving their values or printing along with the calculated data. In the case of the PD method, we can find some advantages. The device is delivered with operating software which allows for automatic measurement of the parameters necessary for the optimization and control of photovoltaic cell manufacturing process with the possibility of saving the data or printing it along with the calculated data, it allows a detailed measurement of selected parameters that can be presented in two formats (graphics, text). It also allows for the setting of the measurement time and it does not require any special pattern for the front electrode. We can find also some drawbacks of this method. For example, it is also a destructive method, and replacement or infusion of parts is costlier than in the TLM method. The front metallization is applied at the front of the silicon solar cells, usually with Ag contacts [20,21] for the surface layer, and full-surface aluminum contacts (Al) with soldering contacts on the back layer of the cell. One of the tendencies is the reduction of the cover value of the front side of the solar cell with metal contacts [22]. 

The goal of this work is to present the results of an investigation into materials co-fired at different temperatures in the infrared belt furnace on three different and independent research stands and to show that the parameters of a solar cell measured with the three methods have differing yet strongly correlated values. The authors’ intention is not to indicate the superiority of any of the presented methods and the results obtained, but to subject them to public and scientific assessment. In addition, the work verified the measurement results for the selected TLM method using the independent research stands (including one non-commercial and two commercial) at three different scientific units. An objective and substantive discussion on this topic will help to find ways to eliminate the discrepancies found and will lead to refinement of the presented measurement methods, or it will be an inspiration towards the proposal of a method that is either new or a compilation of earlier solutions.

## 2. Methods Used

The selection of the metallization parameters of the electrical contacts of the cell reflects the result of measuring the light current-voltage characteristics (I-V), including fill factor (FF) and series cell resistance (*R_s_*). The measurement system can be divided into three basic elements that determine the quality of the measurement, including the light source, the measuring system, and the table and contact probes. The I-V characteristics must be measured under well-defined conditions of a specific radiation spectrum and temperature in the so-called standard test condition (STC). The standards used are class A solar simulators having spectral matching to the AM1.5 spectrum in the range from 0.75–1.25 with admissible inhomogeneity of the illumination ±2% on the surface of the solar cell illuminated by radiation of 1000 W/m^2^ at the cell temperature of 25 °C. The one or two-stage model can be numerically adjusted to the measured I-V characteristics, from which the values of the photocurrent, voltage, dark current, and parallel and serial resistances of the cell are obtained directly. 

**Figure 1:**
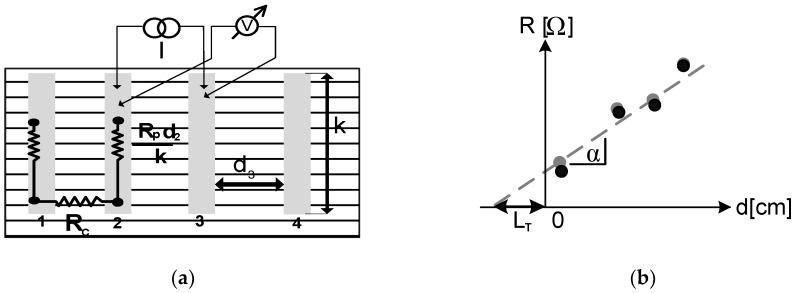
Graphical interpretation of the transmission Line model method (TLM), where the grey point in the graph is the measured resistance and the black point represents the resistance curve adjustment determined using the regression method [23,24]. (**a**) Example of a TLM structure; (**b**)Plot to extract transfer length from the TLM method.

TLM measurements were performed on two independent test stands named as TLM-1 and TLM-2 for deeper verification of the electrical measurement results, in addition to the I-V and PD methods. In the TLM method, the measurement consists of measuring the direct current—I and the voltage—U between a pair of adjacent front electrode paths on the surface of the tested solar cell (1) (Figure 1). A simple linear regression method (2) was used for the measured experimental values obtained from the measurement of the front electrode resistance. The value of the *R_c_* coefficient is obtained from the intersection of the linear regression line with the resistance axis, whereas the value of the *L_T_* current path coefficient is obtained from the intersection of the linear regression line with the distance axis between the electrodes, or it can be calculated from Formula 3. Depending on the value of the *L_T_* coefficient obtained, two conditions for the determination of the correct resistance of the contact were considered. The large values of the *ρ_c_* coefficient lead to the expansion of the current path current, then the width of the electrode finger determines the electrode resistance; therefore, the first condition (4) is satisfied. For low values of *ρ_c_*, the current flows mainly along the edge of the electrode finger, then the resistance does not depend on the electrode finger, so the second condition (5) is satisfied [1,14].

(1) $$R_{T}=\frac{U}{I}\;[\Omega ]$$

where *R_T_* is the measured resistance of the front electrode, I is the specified current value, and U is the measured voltage value between a pair of adjacent front electrode tracks.

(2) $$R_{T}=2R_{c}+\frac{d\cdot R_{p}}{k}\;[\Omega ]$$

where *k* is the length of the front electrode, *d* is the distance between the electrode path lines, *R_c_* is contact resistance, and *R_p_* is the surface resistance of the diffusion layer.

The transfer route of carriers to the contact can be calculated from the formulae [1]:

(3) $$L_{T}=\sqrt{\frac{\rho_{c}}{R_{p}}}\;\mathrm{or}\;L_{T}=\sqrt{\frac{R_{c}}{R_{p}}}$$

Conditions:

(1) If *L_T_* ≥ 2*L* is the specific resistance, then *ρ_c_* is determined by the equation [22]:
  (4) $$\rho_{c}=R_{c}\cdot k\cdot L_{T}\;[\Omega \cdot {\mathrm{cm}}^{2}]$$
  where *L* is the electrode finger width, and *L_T_* is the current flow route;
(2) If *L_T_* < 2*L* is the specific resistance, then *ρ_c_* is determined by the equation:
  (5) $$\rho_{c}=R_{c}\cdot k\cdot L\;[\Omega \cdot {\mathrm{cm}}^{2}]$$

In the case of a non-commercial test stand, the original concept was proposed of the system for testing the electrical properties of the front electrode using the TLM method (mentioned earlier in the paper). In the case of a commercial stand equipped with a Corescan device, the measurement is possible using a measuring probe. Four measurement modes are available for the PD method [25,26] while for the required measurements one should pay attention to the first mapping of the resistance value of the actual contact surface between the emitter and the photovoltaic electrode (Corescan) to optimize the paste during its application to provide the front metallization and controlling of this technological operation [27,28]. In the Corescan unit, a local current is produced as a result of the local lighting while the photovoltaic cell is connected to the external load (resistor). The measurement of the potential difference is possible as a result of the leap between the electrode (metal) and the first point on the adjacent cell substrate. The potential on the front electrode in direct contact with the ground is measured with a metal probe. The cross-section of the manufactured front electrode and the zone of its connection to the ground is then scanned by means of a probe. The device with the relevant software allows for the automatic measurement of selected parameters necessary for optimization and control of the photovoltaic cell fabrication process with the option of the real time recording in textual and graphical (2D and 3D image) formats or their printout along with the calculated data [29]. The obtained results from the software include the contact resistance and contact resistance (Line contact resistance).

The method of contact resistance determination is based on the measurement of the potential leap at the boundary between the metal line and the silicon adjacent to it, while a current flows from the silicon into the metal line. The line contact resistance can be calculated by dividing this potential leap by the current flow into the line (per unit length of the line). The first result refers to the line contact resistance of the front solar cell (6) and it is calculated according to the following formulas presented below: Current flowing through the square (7), current flowing per unit length of the electrode (8), current flowing per unit area of the electrode (9) [29].

(6) $$R_{cl}=C\cdot \frac{V}{I^{\prime }}=C\cdot \frac{V}{d\cdot J}\;[\Omega \cdot \mathrm{cm}]$$

(7) I=J·d·L

(8) $$I^{\prime }=\frac{I}{L}=J\cdot d$$

(9) $$I^{\prime \prime }=\frac{I}{\left(L\cdot w\right)}=\frac{J\cdot d}{w}$$

The second result concerns the specific resistance of the front solar cell electrode (10) and is calculated according to the following formula [1,29]:

(10) $$R_{c}=C\cdot \frac{V}{I^{\prime \prime }}=C\cdot \frac{V\cdot w}{J\cdot d}\;[\Omega \cdot {\mathrm{cm}}^{2}]$$

where *R_cl_* is the line contact resistance, *I*′ is the current flowing through the phase separation surface per unit length of the electrode, *d* is the distance between the electrode path lines, and *V*, the voltage at the front electrode, is measured using a metal probe in direct contact with its surface. The probe scans across the electrode, where *C* is a correction factor, namely the value determined experimentally by dividing *V* at a certain location for the uniform sun illumination, assuming that (*J_sc_* = *J_sc_*, 1_sun_), *V* was found by the Corescan at the same location. *C* ~1.8.

## 3. Research Material

**Figure 2:**
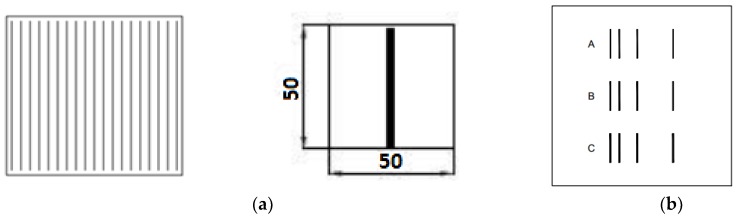
Diagram of contact templates using (**a**) the commercial device for the current-voltage characteristics (I-V) and potential difference method (PD) methods and (**b**) the non-commercial device for the TLM method.

Photovoltaic solar cells and structures were made on the experimental line, according to the following procedure: The surfaces of the Si wafers were texturized in a solution of potassium hydroxide (KOH), and after purification in acids, the donor doping from a source of phosphorus trichloride (POCl_3_) was made. As a result of this process, the n^+^ emitter with the layer resistance value (*R*_sheet_) of 50 Ω/□ was produced. After removing the silicon phosphorous glaze in a 10% hydrofluoric solution (HF) and removing the joint from the edge area, the passivation process was carried out in dry O_2_. Next, the antireflective layer (ARC) from titanium dioxide TiO_2_ was applied. PV505 (solder pads) and PV36A (full electrode) were used respectively to apply the back contacts. The template was designed and made as shown in Figure 2 to measure parameters from the (I-V) characteristic and the resistivity of contacts using the PD and Transmission Line Model method (TLM). The templates were made from 1H18N9 stainless steel. PV19B commercial paste made by Du Pont was used to verify the functional cell parameters by applying it to make the front electrode contacts. After printing the contacts, the structure was subjected to the metallization process in a belt furnace (IR) at process temperature settings in the three successive heating zones of I—530 °C, II—570 °C, and III—*T_M_* at a belt speed of 200 cm/min. One of the variable parameters of the solar cell manufacturing process was the temperature of the metallization process in the *T_M_* range from 870–950 °C. The metallization process was carried out in the natural atmosphere, purified and dried in a filter system.

## 4. Methodology

Measurements of the electrical properties of the fabricated front electrode of photovoltaic solar cells, made using the investigated pastes, were made:

The “I-V” method was used on the sun simulator SS200 of the AAA class from the Photo Emission together with the PV Test Solutions current and voltage measurement system and the I-V Curve Tracer software at 25 °C for the AM1.5 conditions of the solar spectrum. Cell parameters were determined as follows: Open circuit voltage (*V_oc_*), short circuit current (*I_sc_*), fill factor (*FF*), efficiency (*E_ff_*) series resistance (*R_s_*), and parallel resistance (*R_sh_*).

The “PD” method was used on a test stand equipped with the Corescan, which is designed by SunLab (spin-off ECN) and is manufactured and supplied by Mechatronics (under the SunLab license).

The contact resistance (*R_cl_*) and specific resistance (*R_c_*) were determined by the “TLM” method:

➢ On the test stand (labeled as TLM or TLM-1 above) with patent number P.398223. The contact resistance (*R_c_*), specific resistance (*ρ_c_*), and the current flow path (*L_T_*) were determined graphically from the resistance graph measured as a function of distance (*R* = *f* (*d*));
➢ on two independent test stands. The first one was the measuring stand, which was equipped with a Sharp station P-597 made by Unitra-Cemi. Four micromanipulators were used with Karl Süss blade probes and the Keithley SMU device model 2430, operating in DC mode. It was labeled as TLM-2, at the Warsaw Institute, and the second one as TLM-3 at the Wroclaw University of Technology. It was a research stand equipped with Unitra VC-10T meter and Karl Süss needle probes.

## 5. Correlation of the Obtained Results of Electrical Properties

**Figure 3:**
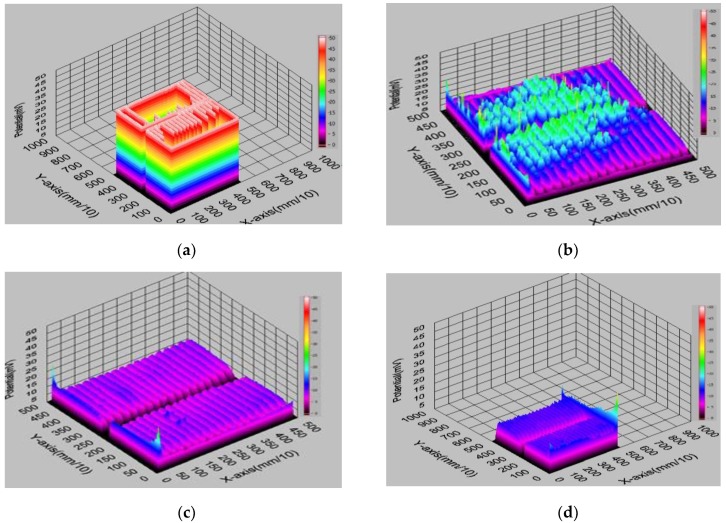
Original measurement printout of the resistance distribution for a cell with a front electrode made of commercial paste and then co-fired in a belt furnace (IR) furnace at (**a**) 840 °C, (**b**) 900 °C, (**c**) 940 ° C, and (**d**) 960 ° C (chosen sample).

The measurement results of the resistance and specific resistance of solar cells are presented in Table 1. Figure 3 presents the results of measurements in the graphical form obtained with PD method using the Corescan device.

Where: *T_M_* is the co-fired temperature, *I_sc_* is the short circuit current of the solar cell, *V_oc_* is the open-circuit voltage of the solar cell, *FF* is the fill factor of the solar cell, E_ff_ is the efficiency of the solar cell, *R_sh_* is the parallel resistance of the solar cell, *R_s_* is the series resistance of the solar cell, I is the current, *J* is the current density, *U_śr_* is the voltage (TLM method), and *U_śr_* is the difference of electrical potentials between two points of the electric circuit (PD method).

The current density of 30 mA/cm^2^ is used in the case of the Corescan device, which corresponds to a current of 750 mA from a cell with an area of 25 cm^2^. In the Corescan device, the current is generated by a small light beam and flows while the photovoltaic solar cell is connected to an external load (resistor). The current flow at the measuring stand P.398223 was forced between the selected pair of neighboring front electrodes and spontaneously generated a potential difference U on them. The measurement is not generated by a beam of light, hence the difference between the currents used on the two test stands. In the case of the I-V method at 840 °C, the highest value of the series resistance was obtained for solar cell 1, and at 920 °C, the lowest value for solar cell 3 was adequately obtained The use of the silver commercial paste made possible the fabrication of a solar cell with a fill factor (*FF*) above 0.70 for solar cells 2 and 4, co-fired sequentially at 900 and 940 °C. With the PD and TLM methods at 940 °C, the lowest resistance and resistance values were obtained for solar cells 9 and 14, and accordingly, the highest value at 840 °C was obtained for solar cells 6 and 11.

As a result, two PD and TLM methods were used to compare the selected electrical parameters of a batch of solar cells with the front electrode from commercial paste PV19B with the metallization temperatures (*T_M_*) of 840, 900, 920, 940, and 960 °C in a belt furnace, which allows us to state that the best properties were obtained for cells (numbers 4, 9 and 14) with the co-firing temperature of 940 °C: *R_cl_* = 2.77 Ωcm, *R_c_* = 27.67 mΩcm^2^ (for the PD method, via commercial test stand), and *R_c_* = 0.4 Ω, *ρ_c_* = 18.34 Ωcm^2^ (for the TLM-1 method, via non-commercial test stand).

The values of the resistance and resistivity parameters of electrical solar cells presented in Table 1 obtained using two test stands and methods using the similar literature formulae show a certain discrepancy. Namely, the desirable lower results were obtained by the TLM method on the P.398223 test stand rather than by the PD method on the Corescan stand.

In order to determine the level of the Pearson’s correlation (r) between the particular methods [29], the data included in Table 1 were used. For the three methods:

- V: Random variables were adopted *R_s_* from the range of cells 1–5: X_Rsi_ = 536.1, 65.7, 63.6, 64.2, 63,9 mΩ;
- PD: Random variables were adopted *R_cl_* from the range of cells 6–10: Y_Rcli_ = 132.57, 3.47, 3.07, 2.77, 3.17 Ωcm;
- TLM: Random variables were adopted *R_C_* from the range of cells 11–15: Z_Rci_ = 1.94, 0.89, 0.53, 0.40, 0.56 Ω.

Based on the calculations, we obtained:

- n=5,
- average value: ${\bar{X}}_{Rs}=158.70\;m\Omega \;,\;{\bar{Y}}_{Rcl}\;=\;29.01\;\Omega \mathrm{cm}\;,\;{\bar{Z}}_{Rc}\;=\;0.86\;\Omega ;$
- standard deviation: ${\mathrm{Sd}}_{X_{\mathrm{Rsi}}}=188.70\;m\Omega ,\;{\mathrm{Sd}}_{Y_{\mathrm{Rcli}}}=51.78\;\Omega \mathrm{cm},\;Sd_{Z_{Rci}}=0.56\;\Omega ,$
- covariance: cov (X_Rsi,_ Y_Rcl_) = 9770.99 mΩΩcm, cov (X_Rsi,_ Z_Rc_) = 101.62 mΩΩ, cov (Y_Rcl,_ Z_Rc_) = 27.89 ΩcmΩ.

The Pearson correlation level (r) was calculated between the particular methods [29]. For the correlation of results for I-V and PD, r = 1.000 was obtained, for I-V and TML, r = 0.958 was obtained, and for the PD and TML methods, r = 0.959 was obtained.

## 6. Verification of the Reliability of the Obtained Results of Measurements Using the TLM Method 

**Figure 4:**
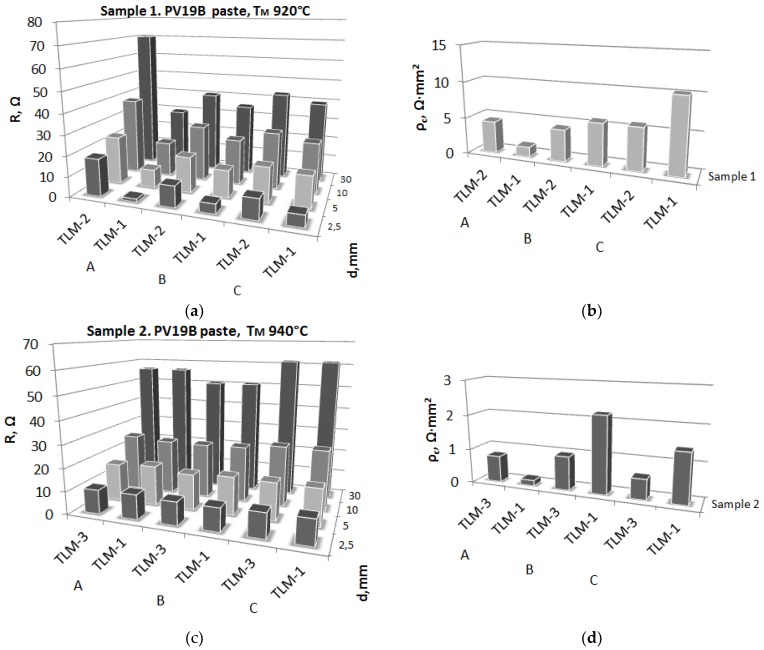
Comparison of resistance (**a**,**c**) and specific resistance (**b**,**d**) among three rows of front electrodes depending on the co-firing temperature for the tested solar cells measured by the TLM-1, TLM-2, and TLM-3 methods in three scientific units (thickness paths A, B, C were 15 μm).

Table 2 and Table 3 present the results of tests obtained using the TLM method at three independent scientific units. Figure 4 shows a graphical interpretation of the resistance and specific resistance of the front electrodes for the investigated pastes on the silicon Si surface made at the above-mentioned test stands.

It was found, based on the comparative analysis of the same photovoltaic cells using the TLM method using independent TLM-1, TLM-2, and TLM-3 test stands, that the measurements of the resistance of the measured *R_T_* of the front electrode are of the same or similar order, and even are identical. It should also be noted that the authors of the presented research results worked independently without informing each other about the results obtained. This also has a significant impact on the reliability and reliability of the research results obtained. We find the occurrence of a small scatter in the calculation of contact resistance (*R_c_*), specific resistance (*ρ_c_*), and current path (*L_T_*), which is determined graphically from the resistance measured as a function of distance (*R* = *f* (*d*)) for TLM-1 measurement stations, and TLM-2 and TLM-3 results from the literature used for measurements and the number of measurements taken.

The above proves the reliability of the obtained results of measurements using the TLM-1 method using the non-commercial P.398223 test stand, as well as the validity of using this method in the photovoltaic cells research.

The comparative analysis shows that the maximum differences in the averaged resistance values of the measured R_T_ front electrode using the TLM method between individual measurement stations are as follows:

➢ TLM-1 and TLM-2, correspondingly, for the whole cell are:
  - PV19B paste: Sample 1 at *T_M_* = 920 °C: 63.2%;
➢ TLM-1 and TLM-3, correspondingly, for the whole cell are:
  - PV19B paste: Sample 2 at *T_M_* = 940 °C: 4.8%.

Differences in the average results of contact resistance and specific resistance between the different methods are, respectively, as follows:

➢ TLM-1 and TLM-2 for sample 1 and *T_M_* = 920 °C: *∆R_C_* = 1.05 Ω and *∆ρ_c_* = 0.93 Ω·cm^2^,
➢ TLM-1 and TLM-3 for sample 2 and *T_M_* = 940 °C: *∆R_C_* = 0.23 Ω and *∆ρ_c_* = 0.52 Ω·cm^2^.

When comparing the obtained results of contact resistance and resistivity using the TLM-1 method with TLM-2 for the solar cell with the front electrode made from PV19B paste with a metallization temperature of 920 °C, it was found that better electrical parameters were obtained with the TLM-2 method, because the contact resistance for the TLM-2 method is lower by 29.6% than in the TLM-1 method (1.05 Ω), and the specific resistance of TLM-2 is lower by 19.2% than in the TLM-1 method (0.93 Ωcm^2^).

Comparing obtained results of contact resistance and resistivity using the TLM-1 method with TLM-3 for the solar cell with the front electrode made from PV19B paste with a metallization temperature of 940 °C, it was found that better electrical parameters were obtained with the TLM-1 method, because the contact resistance for the TLM-1 method is lower by 5.7% than in the TLM-3 method (0.23 Ω), and the TLM-1 specific resistance is lower by 41.3% than in the TLM-3 method (0.52 Ωcm^2^). In summary, the use of the TLM-1 method for comparison with the selected electrical parameters of a batch of solar cells featuring the front electrode made using the commercial paste PV19B with the metallization temperatures of 920 and 940 °C in a belt furnace allows us to conclude that the best properties were obtained for the solar cell at 940 °C: *R_c_* = 4.02 Ω, *ρ_c_* = 1.26 Ωcm^2^ (non-commercial stand).

## 7. Summary 

When comparing the methods used, I-V, PD, and TLM, for each of them the various parameters are determined to assess the electrical properties of the solar cell. In the PD and TLM methods, the same calculation formulae are used. The following results show a high correlation of the methods used and allow for the conclusion that it is enough to apply only one of the three aforementioned methods to determine correctly the parameter that the method allows us to measure. The work verified the measurement results for the selected TLM method using the independent TLM-1 (non-commercial) test stands, TLM-2 and TLM-3 (commercial), at three different scientific units. It should also be noted that the authors of the presented results of the TLM research worked independently, without informing each other about the obtained results. The first statement was made only when writing this article.

## Figures and Tables

**Table 1:** Comparison of selected electrical parameters of a batch of solar cells with the front electrode made from the PV19B commercial paste, measured using three methods.

*T_M_* °C	I-V							PD						TLM			
	Cell no	*I_sc_* mA	*V_oc_* mV	*FF* -	*E_ff_* %	*R_s_* mΩ	*R_sh_* Ω	Cell no	*R*_cl_ Ω·cm	*R_c_* mΩ·cm^2^	*U_śr_* mV	*J* mA/cm^2^	Cell no	*R_c_* Ω	*ρ_c_* mΩ·cm^2^	*U_śr_* mV	*I* mA
840	1	516	546	0.316	3.56	536.1	1.64	6	132.57	1325.67	441.9	30	11	1.94	230.41	849.65	30
900	2	811	591	0.701	13.44	65.7	1.56	7	3.47	34.67	11.57		12	0.89	83.53	535.20	
920	3	804	587	0.660	14.48	63.6	3.98	8	3.07	30.67	10.27		13	0.53	32.95	351.15	
940	4	791	591	0.704	13.17	64.2	0.94	9	2.77	27.67	9.30		14	0.40	18.34	394.95	
960	5	796	587	0.645	12.07	63.9	4.88	10	3.17	31.33	10.43		15	0.56	28.66	488.17	

**Table 2:** Comparison of the results of the measured resistance and specific resistance from three scientific units (where: PV19B paste co-fired in *T_M_*: 920 °C—sample 1, 940 °C—sample 2; A = 0.01. B = 0.02; C = 0.03 cm and denotates path width).

Name of Sample	Name of the Electrode Set	Distance between Electrodes	TLM-2	TLM-1	Unit	Range of Measured Values	
Sample No.	No of electrode paths	d [cm]	R [Ω]	R [Ω]	*k* [mm] *=*	8.00	
					L [mm] =	0.10–0.30	
1	A	0.25	18.36	1.47	*R_c_* [Ω] =	5.52	1.67
		0.5	23.46	8.92	*R_p_* [Ω/□] =	21.41	7.36
		1	38.12	17.09	*L_T_* [mm] =	0.45	0.60
		3	70.18	29.64	*ρ_c_* [Ω·mm^2^] =	4.41	1.34
	B	0.25	10.38	4.76	*R_c_* [Ω] =	2.71	3.62
		0.5	17.51	13.36	*R_p_* [Ω/□] =	17.86	7.88
		1	27.43	22.38	*L_T_* [mm] =	0.49	0.86
		3	40.26	35.23	*ρ_c_* [Ω·mm^2^] =	4.33	5.79
	C	0.25	10.04	5.68	*R_c_* [Ω] =	2.41	4.24
		0.5	17.33	15.66	*R_p_* [Ω/□] =	18.60	8.67
		1	27.77	24.71	*L_T_* [mm] =	0.56	1.08
		3	42.91	39.37	*ρ_c_* [Ω·mm^2^] =	5.79	10.18
Name of sample	Name of the electrode set	Distance between electrodes	TLM-3	TLM-1	*k* [cm] *=*	0.8	
					L [cm] =	0.01–0.03	
2	A	0.25	10.55	10.80	*R_c_* [Ω] =	3.95	4.07
		0.5	16.50	17.50	*R_p_* [Ω/□] =	12.99	12.86
		1	25.45	24.45	*L_T_* [cm] =	0.24	0.56
		3	56.20	56.20	*ρ_c_* [Ω·cm^2^] =	0.77	0.16
	B	0.25	10.10	10.10	*R_c_* [Ω] =	4.14	4.41
		0.5	15.75	16.75	*R_p_* [Ω/□] =	11.34	11.28
		1	24.35	24.88	*L_T_* [cm] =	0.29	0.62
		3	50.25	50.50	*ρ_c_* [Ω·cm^2^] =	0.97	2.20
	C	0.25	10.70	10.90	*R_c_* [Ω] =	3.62	3.58
		0.5	16.45	16.20	*R_p_* [Ω/□] =	14.54	14.54
		1	26.75	26.55	*L_T_* [cm] =	0.20	0.49
		3	61.40	61.40	*ρ_c_* [Ω·cm^2^] =	0.58	1.42

**Table 3:** Comparison of the average results of the contact resistance and specific resistance measured by the three methods.

Sample no.	Symbol of Paste	*T_M_* °C	TLM-1		TLM-2		TLM-3	
			*R_c_*, Ω	*ρ_c_*, Ω·cm^2^	*R_c_*, Ω	*ρ_c_*, Ω·cm^2^	*R_c_*, Ω	*ρ_c_*, Ω·cm^2^
1	PV19B	920	4.60	5.77	3.55	4.84	-	-
2		940	4.02	1.26	-	-	4.25	1.78

## References

[1] Schroder Dieter K. (2015). Semiconductor Material and Device Characterization.

[2] Grover S. (2016). Effect of Transmission Line Measurement (TLM) Geometry on Specific Contact Resistivity Determination. Master’s Thesis.

[3] Geoffrey G., Andrew M.G., Andrew A., Rob J., Zhihao Y., Kristopher O.D. Non-Destructive Contact Resistivity Measurements on Solar Cells Using the Circular Transmission Line Method. Proceedings of the 44th IEEE 2017 Photovoltaic Specialist Conference.

[4] Pyscha D., Mettea A., Glunza S.W. (2007). A review and comparison of different methods to determine the seriesresistance of solar cells. Sol. Energy Mater. Sol. Cells.

[5] https://www.sunlab.nl/wp-content/uploads/2016/12/Corescan-user-manual-V10.02-MRN.pdf.

[6] Panek P., Lipiński M., Ciach R., Drabczyk K., Bielańska E. (2003). The infrared processing in multicrystalline silicon solar cell low-cost technology. Sol. Energy Mater. Sol. Cells.

[7] Gu X., Yu X., Yang D. (2012). Efficiency improvement of crystalline silicon solar cells with a backsurface field produced by boron and aluminum co-doping. Scr. Mater..

[8] Filipowski W., Wrobel E., Drabczyk K., Waczynski K., Kulesza-Matlak G., Lipinski M. (2017). Spray-on glass solution for fabrication silicon solar cell emitter layer. Microelectron. Int..

[9] Filipowski W., Drabczyk K., Wróbel E., Sobik P., Waczynski K., Waczynska-Niemiec N. (2018). Borosilicate spray-on glass solutions for fabrication silicon solar cell back surface field. Microelectron. Int..

[10] Li H., Kim K., Hallam B., Hoex S., Wenham M. (2017). POCl_3_ diffusion for industrial Si solar cell emitter formation. Front. Energy.

[11] Enebish N., Agchbayar D., Dorjkhand S., Baatar D., Ylemj I. (1993). Solar Energy Materials and Solar Cells, An International Journal Devoted to Photovoltaic, Photothermal, and Photochemical Solar Energy Conversion.

[12] Stapiński T., Godlewski M., Jakubowska M., Marszałek K., Pietruszka R., Panek P., Soliński B., Soliński I., Turoń K., Wróblewski G. (2014). Materials and Methods to Optimize the Construction of Cells and Photovoltaic Panels.

[13] Hoornstra J., Van der Heide A., Weeber A., Granek F. New approach for firing optimisation in crystalline silicon cell technology. Proceedings of the 19th European Photovoltaic Solar Energy Conference.

[14] Schroder D.K., Meier D.L. (1984). Solar cell contact resistance—A review. IEEE Trans. Electron Devices.

[15] http://tuttle.merc.iastate.edu/ee432/topics/metals/tlm_measurements.pdf.

[16] Dobrzański L.A., Musztyfaga M., Drygała A., Panek P. (2010). Investigation of the screen printed contacts of silicon solar cells from Transmissions Line Model. J. Achiev. Mater. Manuf. Eng..

[17] Dobrzański A.L., Musztyfaga M., Staszuk M. Test Stand for Electrodes, Especially of the Front Cells of the Silicon Photovoltaic Cell P-398223, dn. 12/11/2014. https://www.polsl.pl/Jednostki/RR6-BRP/Strony/u2014.aspx.

[18] Musztyfaga M. (2011). Laser Micro-Machining of Silicon Elements of Photovoltaic Cells. Doctoral’s Dissertation.

[19] Zhou J., Xu N., Yang H., Zhang Q. (2014). Effect of Ag powder and glass frit in Ag paste on front contact of siliconsolar cells. Procedia Eng..

[20] Hoenig R., Kalio A., Sigwarth J., Clement F., Glatthaar M., Wilde J., Biro D. (2012). Impact of screen printing silver paste components on the space charge region recombination losses of industrial silicon solar cells. Sol. Energy Mater. Sol. Cells.

[21] Mat Desa M.K., Sapeai S., Azhari A.W., Sopian K., Sulaiman M.Y., Amin N., Zaidi S.H. (2016). Silicon back contact solar cell configuration: A pathway towards higher efficiency. Renew. Sustain. Energy Rev..

[22] Pysch D., Mette A., Filipovic A., Glunz S.W.A. (2009). Comprehensive Analysis of advanced solar cell contacts consisting of printed fine-line seed layers thickened by silver plating. Prog. Photovolt. Res. Appl..

[23] (1999). Expression of Measurement Uncertainty Guide.

[24] https://www.sunlab.nl/product/corescan/.URL.

[25] Musztyfaga M., Dobrzański A.L., Rusz M., Staszuk M. (2014). Application examples for the different measurement modes of electrical properties of the solar cells. Arch. Metall. Mater..

[26] Van der Heide A.S.H., Bultman J.H., Hoornstra J., Schonecker A., Wyers G.P., Sinke W.C. Optimizing the front side metallization process using the Corescan. Proceedings of the 29th IEEE Photovoltaic Specialists Conference.

[27] Van der Heide A.S.H., Goris M.J.A.A. Contact optimisation on lowly doped emitters using the corescan on non-uniform emitter cells. Proceedings of the International Conference of Nineteenth European Photovoltaic Solar Energy Conference.

[28] www.mechatronics.nl/support/user/correscan/index.htm.

[29] http://www.naukowiec.org/kalkulatory/korelacja.html.

